# Decorations and Titular Honours

**Published:** 1917-11-24

**Authors:** 


### DECORATIONS AND TITULAR HONOURS.

### Some New Developments and their Relation to the Hospital World.

The question of "honours" in the shape of titular distinctions, orders, medals, ribands, and the rest is evidently becoming a very thorny one. So much, indeed, might have been anticipated in advance, seeing how freely the opportunities and candidates have multiplied. Even in the restricted area of normal days there were doubtless inequalities and grievances, but these were mostly personal or private affairs which had either to be consumed in silence or discussed in a strictly limited circle. Now the field is not only much enlarged but it begins to border on the interests of organisations and corporations, and consequently committees and directors, with a natural eye on the reputation of their enterprises, are driven to watch the position of their officers and servants relative to recognitions which have a spectacular value and some measure of official guarantee. Thus to the rivalry of individuals is added the rivalry of institutions, and as in these circumstances the demand for "justice" may be presented with an impersonal quality it can be sounded without any obvious self-praise or reproach of self-advertisement. The disappointed suitor who has pressed his own case will usually feel it wise to cover with darkness the whole transaction. But when corporate pride is touched there is a ready opportunity for open speech by those who have no direct personal ends to serve, and the opportunity will not invariably be neglected. Hence to a growing extent we may expect to see the distribution of "honours" become subject to a more or less public criticism, and this is just as likely to bewail those that are withheld as to challenge others which are bestowed. Indeed, the process has already begun, and as it has the piquant savour provided by personality, it will be disposed both to attract attention and to engage controversy. What have hitherto been whispers or murmurs in the clubs and around the tea-tables may now be expected to attain the dignity of letters to the editor, or even of authoritative paragraphs.

### New Difficulties.

Nor can this development fail to find a sanction in the argument recently adopted by an authority which speaks here with special force—namely, the House of Lords itself. For if it is a sound principle that only motives capable of public defence can justify public recognition by ribands or what not, the principle must be applied to the whole ground. If budding dukes and earls and baronets and knights must produce a justification for their distinctions, ribands and initials of lesser moment cannot justifiably escape a kindred inquisition, for in essence, and relative to the circles in which they excite ambition, the two groups stand on one and the same platform. The House of Lords formula, indeed, invokes two really tremendous admissions. First, it insists on the necessity for discarding the safe judicial maxim which advises an official voice to provide decisions but not the reasons which determine them. Secondly, it recognises that the creation of the decorated is an affair which concerns actively the undecorated. Inevitably, therefore, if, as is here contended, the principle is one of universal application, the area of admitted and recognised interest is enormously enlarged, and public discussion *pro* and *con* is a natural and unavoidable consequence. The opportunities are increased, the candidates are more numerous, corporate interests are involved, and public attention is invited. It needs but a glance at these considerations to compel the admission that the whole subject has become set with new difficulties.

Another influence which tends towards the same conclusion arises from the fact that, wisely or unwisely, the public has been allowed to contemplate the working of the machinery. Hitherto the mystery behind the scenes has lent a note of dignity and even of austerity to the performance, and while the results have sometimes caused wonder they have never invited open challenge. But recently the working has been laid bare to the public eye, and it has not in every respect excited admiration. A necessary consequence of this will be the cultivation of a more critical inspection, and those concerned with the direction of the apparatus will be conscious of the pressure of a less sympathetic atmosphere. No longer will they feel certain that the crowd will be content to take the goods which the gods provide; in addition to the thunder from the mountain it will be necessary to produce also the still, small voice of reason and justification. The whole process, in short, has passed under new conditions, and these most certainly do not make for smooth or easy or arbitrary working.

### Who Should Make the Choice?

A further consequence of recent developments will doubtless be an inquiry directed to determine where in truth and in fact the power of nomination really resides. That all such recognitions flow from the one and only fountain of honour may be maintained as a pious fiction, but publicity will quicken the desire to penetrate into the active and secret springs. With such a multiplication and extension of the decorated, it becomes openly manifest that the choice, though nominally central, is really determined by a less dignified verdict, and how and by whom this is framed cannot hope to escape speculation. Not only so, but rival claims to this essential power are likely to appear. Indeed, in connection with the award of distinctions to hospital officials rivalry of this order has already made itself heard; and the promise is of more to come. Chairmen and doctors and matrons can surely all put forward a plausible case, and until their several claims are adjusted there may well be lively times. Further, if, as we may learn from the public prints, it may sometimes happen that the views of

**DECORATIONS AND TITULAR HONOURS**—(*cont.*)

every one of these authorities are impartially ignored, there is still no guarantee of peace. Such a position indeed sharpens the desire to know who it was who really spoke the invigorating word, and it gives opportunity to counsellors who regard themselves as neglected to make vocal and public protest. Hence, once again, discussion is provoked, and what was formerly the decision of a supreme choice, and a decision to be received with respectful acquiescence, is now transformed into an opportunity for a controversial and personal debate. "Who should be decorated and who should not?" "Who should decree those who are worthy of adornment?" These are issues which, though not altogether new, have in consequence of recent events taken on an acute character and have become involved in a world of wider interest and keener criticism. What the outcome will be time alone can tell. That there will be changes may be taken as certain, and some of these may easily take on an unexpected shape.

